# Case report: Biallelic variants in *POLR3B* gene lead to 4H leukodystrophy from the study of brother and sister

**DOI:** 10.1097/MD.0000000000030350

**Published:** 2022-08-26

**Authors:** Hengzhou Bai, Dingming Li, Yi Zheng, XiaoHui Jiang

**Affiliations:** a Andrology, West China Second University Hospital, Sichuan University, Chengdu, Sichuan, China; b Key Laboratory of Birth Defects and Related Disease of Women and Children (Sichuan University), Ministry of Education, West China Second University Hospital, Sichuan University, Chengdu, Sichuan, China.

**Keywords:** 4H leukodystrophy, hypomyelination, POLR3B, hypogonadotropic hypogonadism

## Abstract

4H leukodystrophy, one of *POLR3*-related leukodystrophy, is a rare hereditary brain white matter disease caused by the pathogenic biallelic variations in *POLR3A*, *POLR3B*, or *POLR1C*. Hypomyelination, hypodontia, and hypogonadotropic hypogonadism is mainly presented in patients with 4H leukodystrophy.

**Patient concerns:**

Here, we reported the brother and the sister with new compound heterozygous (c.1615G>T and c.165-167del) with various degrees of phenotypes including dysbasia, myopia, dental abnormal, and hypogonadotropic hypogonadism.

**Diagnosis:**

The brother and sister were diagnosed with 4H leukodystrophy.

**Interventions:**

Gonadotrophins treatment of the brother could significantly improve the development of secondary sexual characteristics and genitalia.

**Outcomes:**

This study showed that the same genotype of *POLR3B* may have variable clinical phenotypes in the brother and sister.

**Conclusion:**

The exploration of molecular functions and genetic counseling are crucial for further diagnosis and treatment of *POLR3*-related leukodystrophy.

## 1. Introduction

4H leukodystrophy, a rare hereditary brain white matter disease, was also known as 4H syndrome. The disease manifests as myelin dysplasia leukodystrophies and hypogonadotropic hypogonadism with or without dentition. 4H leukodystrophy was first mentioned and reported 4 cases of patients with dysmyelination of brain white matter, hypogonadotropic hypogonadism, and tooth deformity in 2006.^[1]^ Bernard et al^[2]^ reported that 4H leukodystrophy was caused by *POLR3A* nucleotide variation in 2011. Daoud et al^[3]^ found that *POLR3A* or *POLR3B* nucleotide variation could cause 4H leukodystrophy, and proposed that *POLR3A* nucleotide mutations are more common and frequent in 2013. Based on the clinical data of the brother and sister with 4H leukodystrophy validated by genetical analysis, we summarized the clinical and genetic characteristics of the disease to improve our understanding of 4H leukodystrophy.

## 2. Case reports

### 2.1. Clinical features

**Figure 1.**
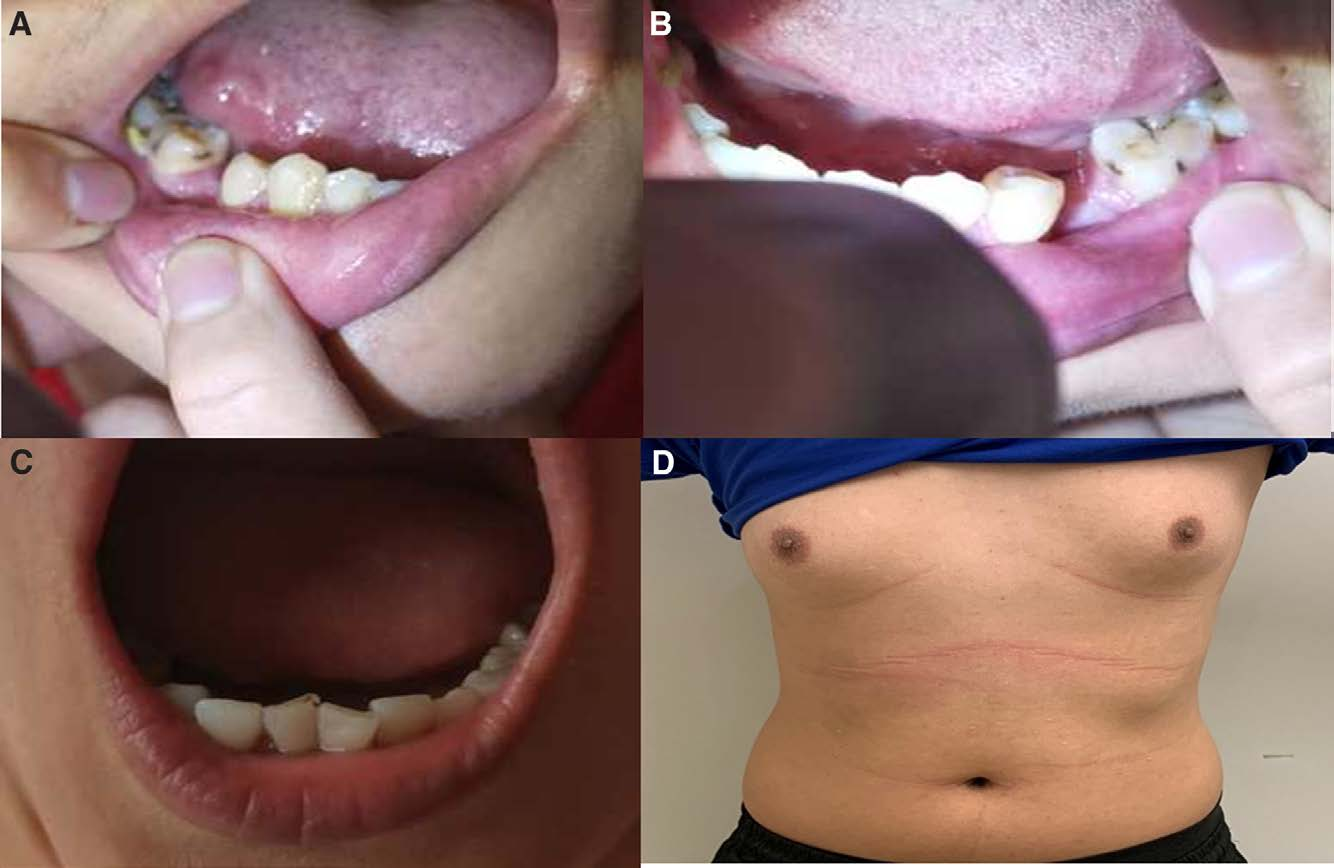
Clinical features of the brother and sister. (A and B) The bilateral hypodontia of brother. (C) Abnormal tooth arrangement of sister. (D) The mammary gland development in brother.

We reported the brother and the sister with gonadal dysplasia diagnosed with 4H leukodystrophy through clinical features and genetic phenotypes. Other family members did not have similar symptoms. The parents revealed that the brother and sister showed gait alterations at the age of 1 year. No treatment was received prior to presentation. At presentation, the brother was 21 years old with 180 cm height, and his sister was 20 years old with 160 cm height. The brother could walk independently with gait clumsiness while the sister was unable to walk without assistance from others. The brother and sister had progressive aggravation of severe myopia and mild strabismus. Hypogonadotropic hypogonadism was obvious in the brother and sister conformed by low levels of follicle stimulating hormone and luteinizing hormone. The brother showed a micropenis and severely reduced testicular volume (single testicle < 3 mL), and the sister had normal mammary gland development and infantile uterus with primary amenorrhea. After the treatment of human chorionic gonadotrophin, 2000 IU twice a week, and human menopausal gonadotrophin, 75I U twice a week, for 3 months, the brother’s single testicular volume was increased from 3 to 8 mL. After 15 months of treatment, the brother had spermatorrhea. In addition, the brother showed hypodontia and the sister only showed abnormal tooth arrangement (Fig. 1A–C). Meanwhile, mammary gland development was discovered in brother (Fig. 1D). Finally, epilepsy, dysarthria, and difficulty swallowing were not detected in the brother and sister. Clinical characteristics were summarized in Table 1.

**Table 1:** Clinical features.

Clinical features	brother	sister
Sex	Male	Female
Age	21	20
Dysbasia	+	+
Age at onset (yr)	Early childhood	Early childhood
Myopia	+	+
Dental abnormalities	+	+−
Hypogonadotropic hypogonadism	+	+
Short stature	−	−
Dysphagia	−	−
Cognitive degression	−	−
Pyramidal signs	−	−
Tremor	−	−
Dysarthria	−	−

### 2.2. Genetic findings

**Figure 2.**
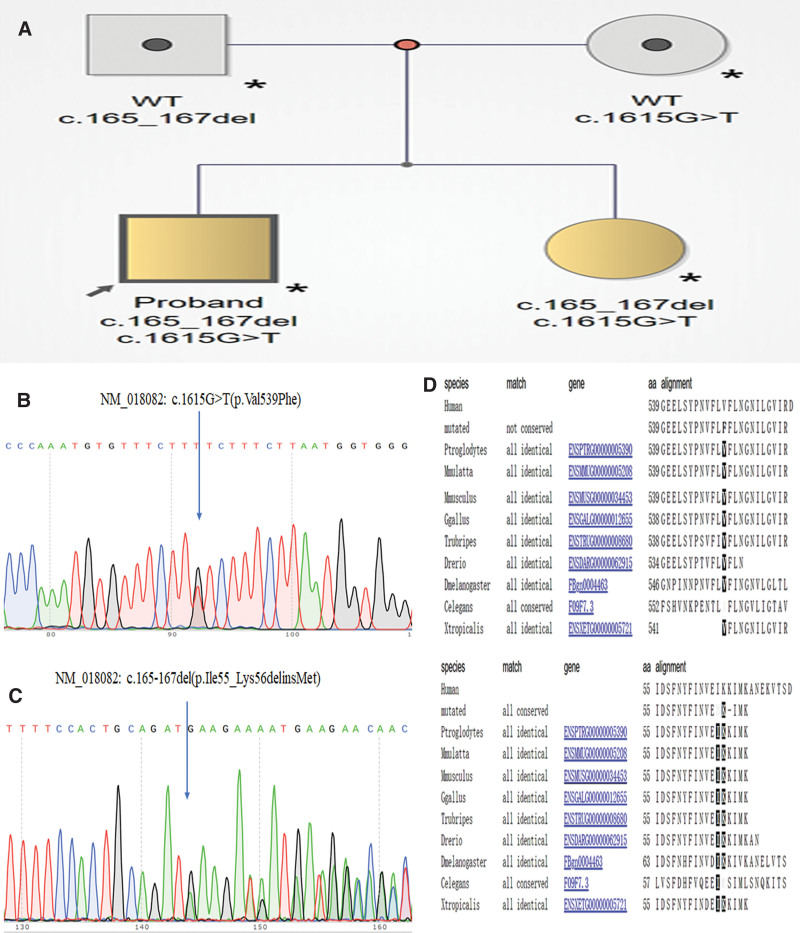
Genetic findings. (A) Pedigree of the investigated proband patients. (B and C) Compound heterozygous variants, NM_018082: c.1615G>T (p.Val539Phe) inherited from their mother and NM_018082: c.165-167del (p.Ile55_Lys56delinsMet) inherited from their father using Sanger sequencing. (D) The positions of mutation were highly conserved in multiple species.

Whole exon sequencing analysis of peripheral blood cells from the brother and sister identified new compound heterozygous variants in the *POLR3B* gene, which were never reported in the Clinvar and Online Mendelian Inheritance in Man. The new compound heterozygous variants, NM_018082: c.1615G>T (p.Val539Phe) (Fig. 2B) inherited from their mother and NM_018082: c.165-167del (p.Ile55_Lys56delinsMet) (Fig. 2C) inherited from their father, were validated using Sanger sequencing (Fig. 2A). The positions of mutation were highly conserved in multiple species (Fig. 2D). Prediction through PolyPhen-2, Protein Variation Effect Analyzer, and Mutation Taster showed both of variations were deleterious.

**Figure 3.**
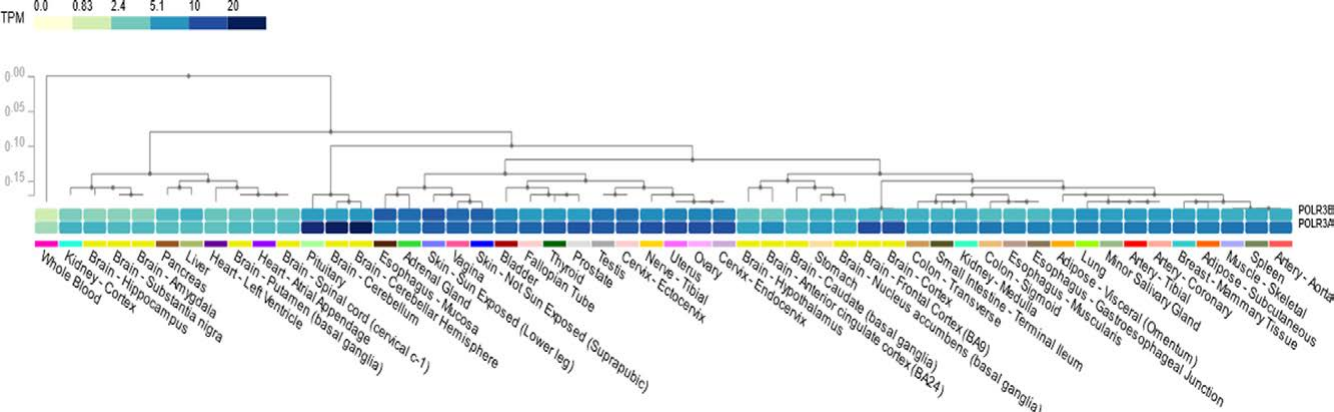
The expression of *POLR3A* and *POLR3B* across multiple tissues in GTEx. GTEx = Genotype-Tissue Expression Project, TPM = transcripts per million.

Structural analysis showed that p.Ile55_Lys56delinsMet was located in protrusion of polymerase III (Pol III) which was critical for binding of the nontemplate strand of the DNA and the interactions with B Double Prime 1, B-related factor 1, and MORF-related gene on chromosome 15-associated factor 1 Homolog, and this variant may lead to the dysregulation of transcription. Besides, p.Val539Phe was located in the lobe of POLR3B protein which together with the POLR3C cleft loop embraced with the nontemplate strand. In addition, we found the expression of *POLR3A* and *POLR3B* were relatively higher in cerebellum across multiple tissues by analyzing The Genotype-Tissue Expression project (Fig. 3).

### 2.3. Magnetic resonance imaging

**Figure 4.**
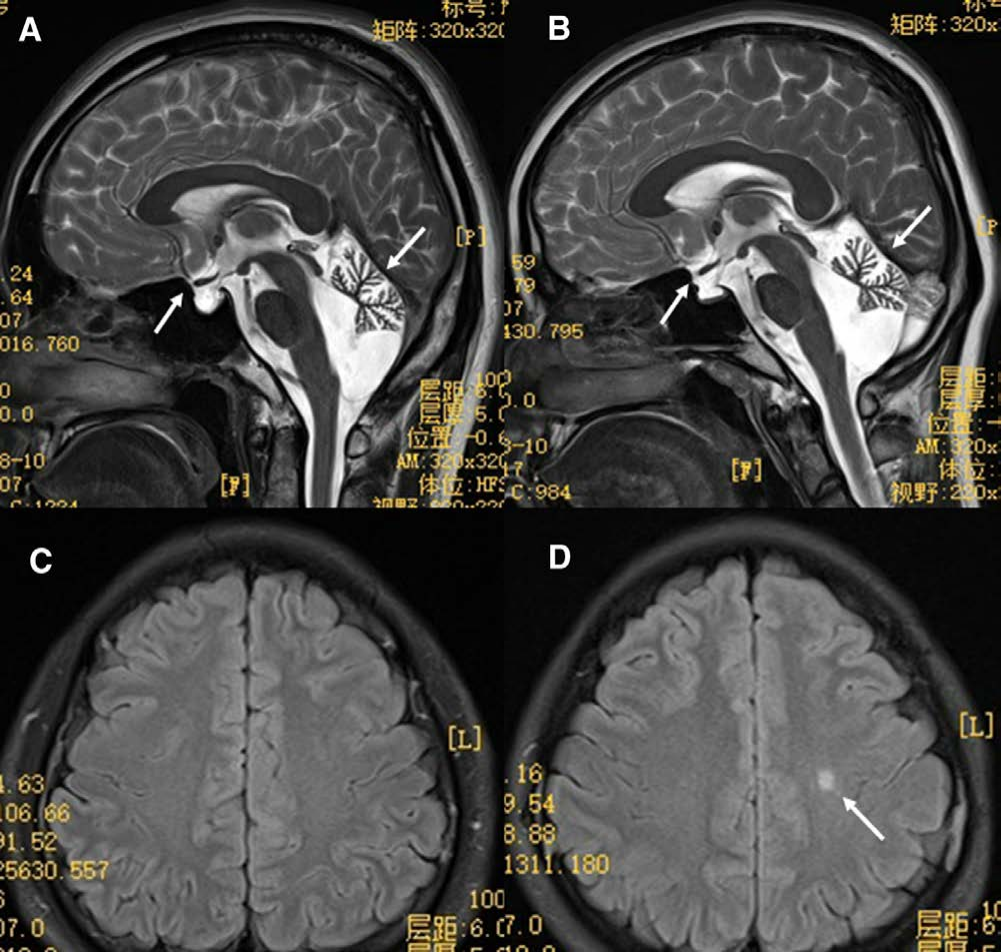
Brain MRI. (A) The cerebellar atrophy and hypophysis dysplasia from brother and (B) from sister. (C) Does not have obvious demyelination in brother. (D) The local demyelination in sister. MRI = magnetic resonance imaging.

The magnetic resonance imaging examination demonstrated that the brother and sister presented obvious cerebellar atrophy and hypophysis dysplasia (Fig. 4A and B). Furthermore, hypomyelination was noticed in the sister consistent with imaging features of 4H leukodystrophy, but not in the brother (Fig. 4C and D).

## 3. Discussion

4H leukodystrophy, one of *POLR3*-related leukodystrophy, is a rare autosomal recessive disease characterized by hypomyelination, hypodontia, and hypogonadotropic hypogonadism. The mutations in *POLR3A* and *POLR3B* are the most frequently reported etiology for 4H leukodystrophy. In addition, Gauquelin et al^[4]^ reported 23 patients with RNA Pol III-related leukodystrophy caused by biallelic *POLR1C* variants in a cross-sectional observational study involving 25 centers worldwide. Recently, a multicenter retrospective study including 150 patients with 4H leukodystrophy demonstrated that 56 (37.3%) patients carried the mutations in *POLR3A*, 81 (54%) in *POLR3B*, and 13 (8.7%) in *POLR1C*.^[5]^ These mutations included homozygous mutation, compound heterozygous mutations, intronic mutations, and large exonic deletions. The distributions of mutation pattern around world are very variable, such as the intronic c.1909 + 22G>A variant in *POLR3A* is mainly distributed in Europe^[6]^ and only several *POLR3A* related leukodystrophy were reported in China.^[7–9]^ The mutations of our patients were not reported previously and described in the Genome Aggregation Database with an allele frequency of 0.000032% for c.1615G>T (rs1171962004) and 0.00008% for c.165-167del (rs745721628) only in Asian (www.ncbi.nlm.nih.gov/snp/). So, this compound heterozygote in the brother and sister is never reported in other countries in the world.

*POLR3A* and *POLR3B* encode the first and second subunits of RNA Pol III respectively, which is an essential eukaryotic RNA polymerase responsible for the synthesis of several types of noncoding RNAs (ncRNAs), transfer RNAs (tRNAs), 5S ribosomal RNA (rRNA), and U6 small nuclear RNA. Pol III complex consists of 17 units in which *POLR3A* and *POLR3B* form the catalytic center. Although the crystal structure of RNA Pol III and interaction between complex and target DNA have been identified, the molecular mechanism of the leukodystrophy pathophysiology still lack research. We found that the cerebellum expresses the almost highest level of *POLR3A* and *POLR3B* across multiple tissues by analyzing the RNA-Seq data from The Genotype-Tissue Expression project. Choquet et al^[10]^ reported the first transgenic mice with biallelic mutations in *POLR3A* which failed to display hypomyelination or cerebellar atrophy and this absence of a phenotype in mice may be due to the diversity of species, mutation location, and environmental stressors. Afterwards, Choquet^[11]^ developed a cell model with *POLR3A* mutation in which the expression of target gene related to generation of CNS myelin was decreased. Finally, the mice carrying the *POLR3B* mutation was also established and homozygosity for this mutation was embryonically lethal.^[12]^ Since *POLR3A* and *POLR3B* play an important role in the development of the central nervous system, proper models of different mutation location of *POLR3* is crucial for the exploration of pathophysiological mechanisms.

4H leukodystrophy has a wide spectrum of clinical variability, including neurological and nonneurological manifestations. Wolf et al^[13]^ revealed that more than half of patients needed wheelchair assistance as adults. Patients with 4H leukodystrophy also presented delayed puberty or primary amenorrhea, including 27/33 patients with *POLR3A* (81%) and 20/29 patients (69%) with *POLR3B* mutations.^[13]^ Furthermore, 20% patients had vertical gaze palsy, 19% patients with epilepsy, 87% with dental abnormalities, 72% with hypodontia, 87% with myopia, and 51% with short stature.^[13]^ Moreover, almost all of these patients had hypomyelination in magnetic resonance imaging and *POLR3B* patients had cerebellar atrophy. Compared with our results, the brother and sister had the typical dysbasia, severe myopia, dental abnormal, endocrine dysfunction, and cerebellar atrophy. However, there were only mild hypomyelination in the sister as well as not short stature in the brother and sister. This might be explained by a viewpoint that diffuse hypomyelination was not an obligatory feature for 4H leukodystrophy that had reported in previous study.^[14]^ Considering ambulation without wheelchair and normal cognition in our patients, we also thought clinical phenotype of *POLR3B* mutation patients were lighter than *POLR3A* mutation which was also in keeping with the opinion from previous research.^[13]^

4H leukodystrophy belongs to a rare genetic disease and expert consensus for treatment is still undocumented. The parents came to our department to seek the improvement of sexual development for the brother. The brother presented with low baseline follicle stimulating hormone and luteinizing hormone levels, as well as a lack of response to stimulation with gonadotropin-releasing hormone. Of course, treatment of hypogonadotropic hypogonadism may induce a growth spurt, which may aggravate difficulty in walking in patients with short stature.^[5]^ Given the normal height of our patients and consent from parents, the brother received the combination treatment of human chorionic gonadotrophin and human menopausal gonadotropin with well therapeutic efficacy. In addition, a large-scale whole exome sequencing had identified 4 individuals with *POLR3B* mutations in a large cohort of subjects with hypogonadotropic hypogonadism.^[15]^ Kunii et al^[16]^ had identified pathogenic mutations in 8 of the 60 adult leukoencephalopathy patients (one subject with *POLR3A* mutation) in Japan using a gene panel including 55 leukoencephalopathy-related genes. In view of this, genetic counseling should be recommended for these patients if they have a request for fertility. Unfortunately, the sister was not treated.

## 4. Conclusion

In conclusion, the brother and the sister with new compound heterozygous variants in *POLR3B* had typical leukodystrophy symptom. The same genotype of *POLR3B* may have variable clinical phenotypes in the brother and sister. The exploration of molecular function and genetic counseling are crucial for further diagnosis and treatment of *POLR3*-related leukodystrophy.

## Author contributions

HB, DL, and XJ contributed to the conception, data interpretation, and preparation of the manuscript. YZ contributed to the clinical data of the patients described in this manuscript.

**Conceptualization:** Hengzhou Bai, XiaoHui Jiang.

**Data curation:** Hengzhou Bai, Yi Zheng.

**Investigation:** Dingming Li, XiaoHui Jiang.

**Methodology:** Dingming Li, Hengzhou Bai, XiaoHui Jiang.

**Project administration:** XiaoHui Jiang.

**Writing – original draft:** Hengzhou Bai.

**Writing – review & editing:** Hengzhou Bai, Dingming Li, XiaoHui Jiang, Yi Zheng.

## Acknowledgements

The authors acknowledge with thanks the cooperation of the patients.
